# Skeletal effects of eccentric strengthening exercise: a scoping review

**DOI:** 10.1186/s12891-023-06739-6

**Published:** 2023-07-25

**Authors:** Harshvardhan Singh, Bethany A. Moore, Roshita Rathore, William R Reed, William R. Thompson, Gordon Fisher, Donald H. Lein, Gary R. Hunter

**Affiliations:** 1 Department of Physical Therapy, University of Alabama at Birmingham, Birmingham, AL US; 2 Department of Nutrition Sciences, University of Alabama at Birmingham, Birmingham, AL US; 3 Department of Physical Medicine and Rehabilitation, Heersink School of Medicine, University of Alabama at Birmingham, Birmingham, AL US; 4 Department of Physical Therapy, Indiana University, Indianapolis, IN US; 5 Department of Kinesiology, University of Alabama at Birmingham, Birmingham, AL US

**Keywords:** Eccentric, Exercise, Bone, Older, Load

## Abstract

**Background:**

Conventional progressive concentric strengthening exercise (CSE) to improve bone mineral density (BMD) and bone mineral content (BMC) may not be feasible for populations with chronic musculoskeletal and/or metabolic conditions, such as osteoporosis or obesity. Muscle lengthening exercise, also known as an eccentric strengthening exercise (ESE), may have a special utility for those populations due to greater force generation versus CSE. In fact, greater mechanical loading can be induced on bone at lower resistance levels with ESE. However, effects of ESE on BMD and BMC are unclear. Thus, the purpose of this review was to interrogate the effects of ESE on BMD and BMC.

**Methods:**

A literature review was conducted between January 1995 and April 2022 focusing on randomized controlled trials investigating the effects of ESE on BMD and/or BMC in humans. Terms covering the domains of exercise, bone, and populations were searched on PubMed, CINAHL, and Scopus. The methodological quality of each interventional study was rated using Physiotherapy Evidence Database (PEDro) scale. Cohen’s *d* was calculated to determine the magnitude of the effects of ERE on site-specific outcome measures of BMD and/or BMC.

**Results:**

Out of 1,182 articles initially found, a total of seven full length articles met our inclusion criteria. Of the seven studies, most of the interventions were performed in young (*n* = 5, PEDro = 5–7) versus middle-aged (*n* = 1, PEDro = 4) or older (*n* = 1, PEDro = 6) adults. BMD and BMC generally improved due to ESE; however the effects of ESE on BMD and BMC were non-homogenous. Effect size (*d*) ranged from 0.10–0.87 in young adults while it was 1.16 in older adults. Effect size (*d)* could not be calculated for the middle-aged adult study due to critical methodological limitations of the intervention.

**Conclusions:**

Large variability exists for the effectiveness of ESE on BMD/BMC across the human life spectrum. The benefits of ESE on BMD holds promise but rigorous studies are lacking. Further research is needed to examine if the dose, mode, age, and sex-specificity dictate effects of ESE on BMD/BMC.

## Introduction

Mechanical loading on bone induced by strong muscle contractions generates potent osteogenic signals, [1] thus driving mechanical adaptation of bone [2, 3]. A popular technique to generate osteogenic mechanical loading on bone is conventional progressive resistance strengthening exercise (CSE) training programs. CSE can increase: 1) bone mineral content (BMC), 2) bone mineral density (BMD) of the hip and the lumbar spine in older men with low bone mass; [4] 3) BMD and BMC of the lumbar spine and femoral neck in women across pre- to post-menopausal stages; [5] and 4) improve bone architecture in young population [6]. Previous findings also show that muscle strength is positively associated with, and predictive of, BMD in older adults [7]. Notably, BMD and BMC are independent, robust predictors of future fractures [8]. Osteogenesis is stimulated and bone catabolism is reduced via direct and indirect effects of mechanical loading. Increased strain and pressure on osteocytes via increased fluid flow in the lacunar-canalicular network is one of the main direct pathways of anabolic stimulus on bone [9]. Whereas increased expression of local growth factors and muscle turnover proteins such as insulin-like growth factor-1 and fibroblast growth factor 2 stimulate bone indirectly [10, 11].

CSE is based on the determination of one repetition maximum (1RM), which is the maximum load a muscle can lift concentrically, or during its shortening phase, which is typically an open kinetic chain exercise. Typically, CSE programs require participants to lift 70-85% of 1RM to achieve beneficial effects on BMD [12]. Although there is emerging evidence that individuals with low bone mass may safely perform high-intensity CSE with beneficial effects on BMD [13], there is a lack of sufficient data on the effects of high-intensity CSE on BMD in populations with chronic musculoskeletal conditions, such as older adults with osteoporosis, who may be frail and have low exercise tolerance [14]. High-intensity CSE, which is mainly dictated by concentric strength, also places increased demands on other physiological systems such as the cardiovascular system [15] which may not be optimal in populations with low exercise tolerance. Increased risk of injuries with CSE, [16] especially with 1RM testing [16, 17] has been reported resulting in calls for use of caution when performing high-intensity CSE in individuals with chronic conditions, who may have a compromised ability to lift heavy loads. These factors may explain the dearth of data on the skeletal effects of high-intensity resistance training in individuals with chronic musculoskeletal conditions and low exercise tolerance such as older populations or individuals with osteoporosis.

Muscle lengthening exercise, also known as eccentric strengthening exercise (ESE), is another technique of exercise training where muscle lowers a load under resistance. An example of ESE is slowly lowering oneself from standing to sitting while wearing a weighted vest, which is an example of closed kinetic chain exercise. High stretch forces are created during ESE which can exert significant mechanical loading on the skeletal system, thereby providing an anabolic bone stimulus. This is corroborated by the fact that ESE increases BMC and BMD in young [18–21] and older adults [22]. ESE may have a special utility for older adults due to 1) lower decline in eccentric versus concentric strength with aging, [23] and 2) greater force generation (up to ~30%) versus concentric strength at the same relative intensity [24]. Due to age-associated increases in passive stiffness, [20] connective tissue, [25] viscoelastic forces, [25] as well as a lower rate of eccentric strength loss versus concentric strength, [23, 26] ESE may have a special utility to create greater mechanical load in older adults. Moreover, ESE has lower cardiometabolic demand than CSE [27] which makes ESE safer for individuals with chronic conditions. In addition to indirect mechanical loading on bone, ESE can also generate direct mechanical loading on bone if applied as closed kinetic chain exercise. This is important because direct mechanical loading on bone can positively influence shear stress, direct strain, and pressure on osteocytes by increased fluid flow in the lacunar-canalicular network leading to osteogenesis [9]. Thus, it can be postulated that ESE holds promise as an efficient treatment for skeletal deficits in populations with chronic musculoskeletal conditions. To know if indeed ESE could be applied as an effective intervention within skeletal rehabilitation paradigm, there is a need to review the evidence in the published literature regarding the effects of ESE on BMD and BMC to better inform exercise scientists, health care practitioners, physical therapists, and rehabilitation professionals. Such information can help design novel evidence-based rehabilitation protocols for maximal treatment outcomes for individuals with chronic musculoskeletal conditions.

Thus, the purpose of our review was to methodically examine the available evidence on the effects of ESE on BMD and BMC in young, middle-aged, and older adults. We have reported our review based on articles found between the years 1995 - 2022. To our knowledge, there are no reviews on the effects of ESE on BMD and BMC in humans. Due to the very low number of intervention studies, lack of consistency with the ESE technique, and unique protocols employed in each intervention study, we could not perform meta-analyses. Our review provides the state of current evidence regarding the potency of ESE to increase BMD and BMC in humans while also providing recommendations for clinical practice and directions for future research in this area.

## Methods

A review of randomized clinical trials that included ESE intervention was performed.

### Search strategy

**Fig. 1:**
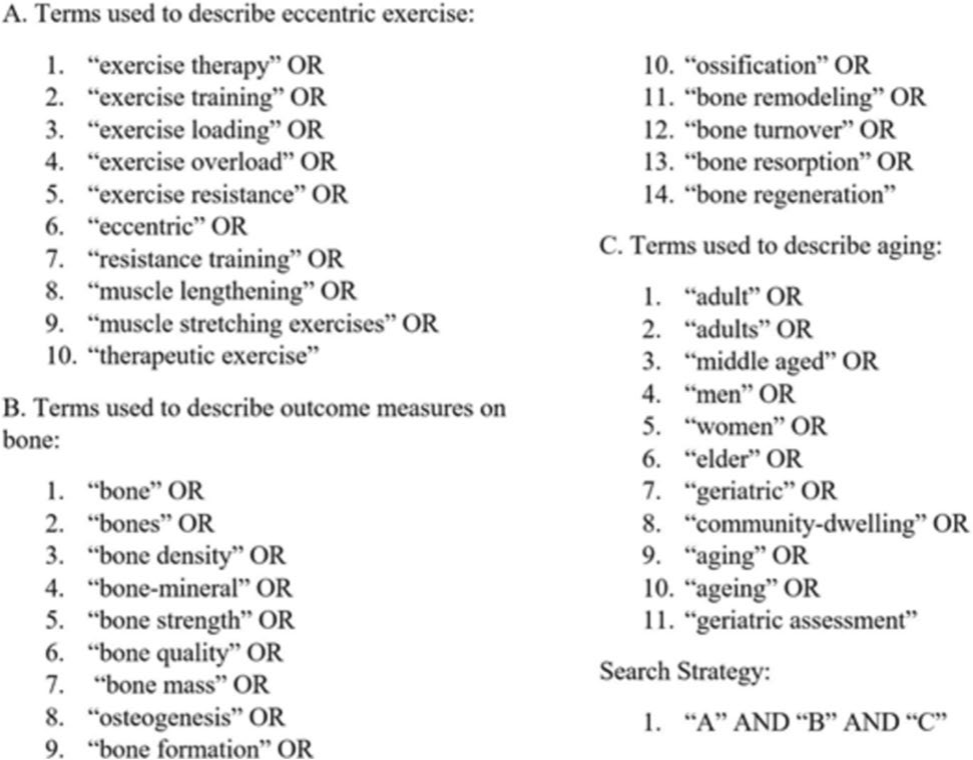
Search terms and strategy (Database used: PubMed, Scopus, CINAHL)

Our literature search included terms as shown in Fig. 1 to examine articles from the following databases: PubMed, Cumulative Index to Nursing and Allied Health Literature (CINAHL), and Scopus. Our search period for articles ranged from January 1995 to August 2022. We also consulted a research reference librarian who works at the University of Alabama at Birmingham to verify the article list using the same search terms.

### Inclusion and exclusion criteria

Studies selected for inclusion met the following criteria: 1) were randomized controlled trials, 2) were written in English and published between January 1995 and April 2022, 3) included an eccentric exercise intervention, 4) included outcome measures of BMD and/or BMC, and 5) included participants 18 years of age or older. Studies were excluded if they were not in English or employed combined interventions which were not uniquely eccentric exercise.

### Selection of articles and data extraction

**Fig. 2:**
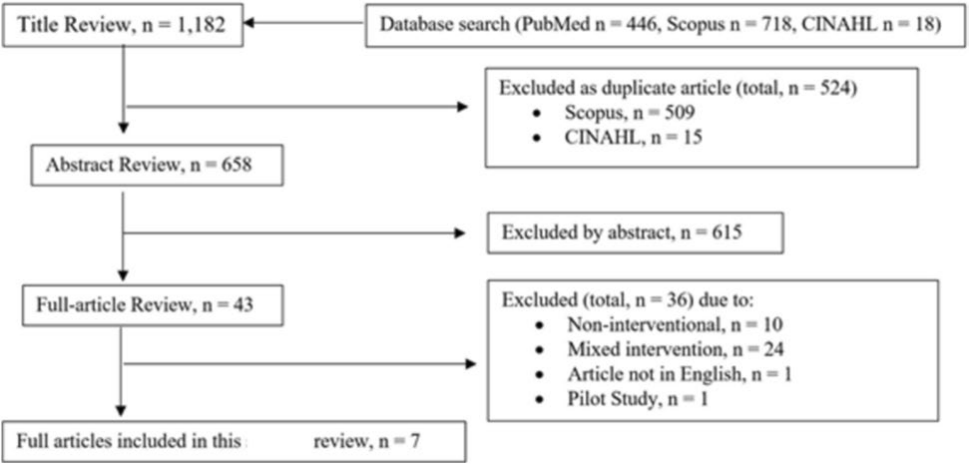
Data extraction; CINAHL, Cumulated index to nursing and allied health literature

Figure 2 displays our procedure for the selection of articles and data extraction. Two independent reviewers (BM, RR) assessed the titles of all the articles (*n* = 1,182) found from the aforementioned three databases and searched using a standardized form, created specifically to determine studies’ relevance to this review. If the relevance of the article could not be obtained by the title, the article’s abstract was consulted. Any discrepancies between the two reviewers were brought to a third reviewer (HS) for discussion until a final consensus was obtained. After a consensus had been obtained, the pertinent article abstracts (*n* = 658) were read by the two reviewers (BM and HS).

Each reviewer independently assessed identified abstracts against the previously mentioned inclusion/exclusion criteria and then met to discuss and obtain consensus on the relevancy of the articles’ abstracts. Upon obtaining consensus, the full-length articles (*n* = 7) were redistributed among the reviewers for complete review and data extraction. Each reviewer ascertained the study’s principal author, a description of the intervention, population characteristics including sex, the intervention period, any secondary interventional procedure that was performed, and the outcome measures of each full-length article. Reviewers then met to discuss any disagreements from the data extraction and to consolidate data. No disagreements were noted between the reviewers for the final 7 full-length articles.

Next, reviewers rated the quality of all the 7 final full-length articles using ‘The Physiotherapy Evidence Database’ (PEDro) scale (Table 1), which is a validated measure assessing the methodological quality of clinical trials [28]. PEDro scale uses a list of 10 scored questions pertaining to the methodological quality of clinical trials. The scored 10 items are as follows: 1) random allocation of subjects, 2) concealed allocation, 3) similarity at the baseline, 4) blinding of subjects, 5) blinding of the tester, 6) blinding of all assessors, 7) one key outcome obtained from > 85% of original subjects, 8) intention-to-treat analysis, 9) between-group comparisons for a minimum of 1 key outcome, and 10) point and variability measures for a minimum of 1 key outcome. Each of the 10 questions in PEDro scale are scored either 1 or 0 based on the information provided in the study. A PEDro score of ≥ 6/10 indicates a moderate to high-quality study. Reviewers also examined the magnitude of the effect/effect size of each study intervention by calculating Cohen’s *d*. Effect size conveys the magnitude of change in outcome measures due to intervention [29] and thus is useful for rehabilitation scientists, health care practitioners, and clinicians in designing optimal evidence-based exercise protocols for specific populations or conditions [30].

**Table 1:** Study characteristics and quality rating

**Study**	**Design**	**PEDro Score**	**Sex**	**Sample Size**	**Age** **(mean ± SD in years)**	**Age Group**
Alfredson et al. (1998) [31]	RCT	4	M/F	*n* = 14; 12 M, 2 F	44.2 ± 7.1	Middle
Hawkins et al. (1999) [20]	RCT	6	F	*n* = 16	20.8 ± 1.17	Young
Schroeder et al. (2004) [21]	RCT	7	F	*n* = 37	24.4 ± 1.9	Young
Miller et al. (2007) [32]	RCT	7	F	*n* = 54	20.0 ± 1.7	Young
Nickols-Richardson et al. (2007) [19]	RCT	7	F	*n* = 70	20.1 ± 1.4	Young
English et al. (2014) [18, 19]	RCT	5	M	*n* = 40	34.9 ± 7	Young
Chen et al. (2017) [22]	RCT	6	F	*n* = 30	66.4 ± 6.8	Old

*RCT* Randomized controlled trial, *PEDro* The Physiotherapy Evidence Database, *RCT* Randomized control trial, *M* Male, *F* Female

PEDro scores of ≥ 6/10 indicates moderate to high quality study

### Statistical analysis

The magnitude of the effect of the eccentric exercise intervention on BMD and BMC outcome measures were site-specific and shown as Cohen’s *d* for 6/7 studies (no effect size was calculated for Alfredson et al. [31] because of a lack of pre-intervention data from the intervention group [31] Table 2). Cohen’s *d* was calculated only for those skeletal sites where significant statistical differences were reported due to ERE intervention. The magnitude of the effect size was defined as *d* = 0.20 small, *d* = 0.50 medium, and *d* = 0.80 large [30]. An effect size calculated as small suggests minimal to no effect of the study intervention on outcome measures while a large effect size shows marked changes in the outcome measures due to the study intervention.

**Table 2:** Descript ion of studies

Study	Exercise Intervention Description	Duration	Outcome Measures	Results
Hawkins et al. (1999) [20]	1. Subjects were randomly selected to train one leg CSE and one ESE 2. CSE leg performed 3 sets of 4 1-repetition maximum 3. ESE leg completed 3 sets of 3 1-repetition maximum 4. Each set was performed with continuous extension/flexion movement and a 1-min recovery was taken between sets	18 weeks; 3 non-consecutive days/week	BMD of the whole leg, mid-thigh, and total BMD	1. ESE training significantly ↑ mid-femur BMD 2. Post mid-femur BMD for the ESE group was significantly greater than the controls; neither CSE or ESE training ↑ whole leg segment BMD 3. No significant differences between exercise or control participants in pre- and post- total BMD or the hip BMD
Schroeder et al. (2004) [21]	1. 6 supervised exercises performed with trainer present to lift load through CSE portion of exercise 2. High–intensity group (*n* = 14) 3 sets of 6 reps at 125% CSE max 3. Low-intensity group (*n* = 14) 3 sets of 10 reps at 75% CSE max	16 weeks; 2 days/week	BMC and BMD of the spine, proximal femur mid-femur, and total body	1. No change in BMD was noted 2. Changes in BMC of the spine in LRT group (0.855 ± 0.958 g)
Miller et al. (2007) [32]	1. Participants were randomized into CSE (*n* = 32) or ESE (*n* = 22) 2. Starting set of 6 maximal reciprocal elbow extension and flexion reps with non-dominant arm 3. An additional set was added until 5 (maximal) total sets were performed each session	20 weeks; 3 non-consecutive days/week	BMD and BMC of the trained and untrained ulna	1. Isokinetic training ↑ ulnar BMC and BMD from baseline when compared to untrained ulna 2. ↑ ulnar BMC and BMD only seen in CSE group when controlling for changes in untrained limb
Nickols-Richardson et al. (2007) [19]	1. Participants were randomly assigned to either CSE or ESE exercise modalities and trained using only their non-dominant arm and leg 2. During week one of the study period each participant completed one set of six reciprocal knee and elbow extension and flexion repetitions at 60°/s using only their designated modality 3. From week one of the study until the fifth the number of sets/week was ↑ by one until a total of five sets was completed in one session 4. After week 5, the sets remained at 5 for the duration of the study 5. Torque was not controlled for	20 ± 1 week; 3 non-consecutive days/week	BMC and BMD of the total body, non-dominant and dominant total proximal femur, distal tibia, and total forearm	1. ESE training significantly ↓ distal tibia BMC of the trained leg 2. Significant ↑ in total forearm BMD and BMC were seen in both groups when effects of variation in the untrained limb were controlled for
English et al. (2014) [18, 19]	1. All participants trained with the same CSE load, but ESE loads were prescribed differently for each of the five groups (0%, or concentric training only, 33, 66, 100, or 138% of the CSE load) 2. Each week of training followed this format: exercise day one = heavy day, exercise day two = light day (~ 10% of 1-RM less than day 1), exercise day three = moderate day (~ 5% of 1-RM less than day 1)	12 weeks total; 3 weeks pre-training, 8 weeks training and 1-week post-training; training was 3 days/week	BMD of the whole body, lumbar spine, and hip	1. The total hip, femoral neck, and hip intertrochanter BMD were unchanged in all groups 2. After training 138% group ↑ greater trochanter BMD 3. The total lumbar BMD ↑ in all groups except 100% group, but no between group differences were found
Chen et al. (2017) [22]	1. Participants were split into two group ASW or DSW 2. At week one, participants ascended or descended the 110 stairs (1 rep) twice 3. Volume was increased as the study progressed so that by week 12 each participant completed 24 reps/session 4. An elevator was used to carry participants either up or down the stairs depending on group assignment	12 weeks; 2 days/week	Calcaneal BMD of the right heel	1. BMD ↑ by 6.1% in DSW group 2. No significant change in ASW group was noted

*Abbreviations:* *CSE* Concentric strengthening exercise, *ESE* Eccentric strengthening exercise, *BMD* Bone mineral density, *BMC* Bone mineral content, *RM* Repetition maximum, *ASW* Ascending stair walking, *DSW* Descending stair walking

## Results

Seven randomized clinical trials [18–22, 31, 32] were included in our final analysis. Out of seven studies, five studies were done in young adults, [18–21, 32] one in middle-aged adults, [31] and one in older adults [22]. Out of the five studies done in young adults, four studies had only female participants [19–21, 32] while the remaining study involved male participants [18] only. The middle-aged adult study by Alfredson et al [31] was comprised of men and women, whereas the older adult study by Chen et al. [22] had only women participants. We focused on two outcome measures: BMD and BMC. Most of the included studies in this review paper focused on the skeletal sites of the lower extremity and the spine [18, 20–22, 31] while two studies investigated skeletal sites of the upper extremity [19, 32]. Outcome summaries of each study are illustrated in Table 3. Effects of ESE on BMD and BMC per different age groups are as follows:

**Table 3:** Magnitude of difference, calculated as Cohen’s *d,* of variables BMC and BMD for all included studies

Study	Site	Variable	Pre		Post		Sample Size (n)	*P* value	Effect Size (Cohen’s *d*)^a^
			**Mean**	**SD**	**Mean**	**SD**			
Hawkins et al. (1999) [20]	Mid Femur	BMD	1.429	0.16	1.486	0.18	8	*p* < 0.05	0.33
Schroeder et al. (2004) [21]	Spine, Low-intensity RT	BMC	54.5	6.9	55.4	6.5	14	*p* = 0.05	0.15
Miller et al. (2007) [32]	Trained Ulna	BMC	4.80	0.62	4.91	0.66	22	*p* < 0.001, *limb x time*	0.17
	Untrained Ulna	BMC	4.97	0.76	5.05	0.78		*p* < 0.001	0.10
	Trained Ulna	BMD	0.527	0.038	0.535	0.044		*p* < 0.001	0.19
Nickols-Richardson et al. (2007) [19]	Total Proximal femur	BMD	0.927	0.018	0.938	0.018	33	*p* < 0.001	0.61
	Total proximal femur	BMC	29.64	0.84	29.95	0.83		–	0.37
English et al. (2014) [18, 19]	Greater trochanter, 138% group	BMD	0.778	0.04	0.784	0.04	8	*p* < 0.05	0.15
	L1, 33% group	BMD	1.090	0.02	1.104	0.03			0.55
	L1, 138% group	BMD	1.003	0.04	1.167	0.03			0.7
	L2, 33% group	BMD	1.143	0.03	1.167	0.03			0.8
	L2, 138% group	BMD	1.092	0.05	1.120	0.04			0.7
	L3, 33% group	BMD	1.119	0.04	1.136	0.04			0.43
	L4, 0% group	BMD	1.186	0.03	1.212	0.03			0.87
	L4, 33% group	BMD	1.087	0.04	1.114	0.04			0.68
	L4, 66% group	BMD	1.050	0.04	1.080	0.04			0.75
	Total Lumbar, 0% group	BMD	1.148	0.03	1.163	0.03			0.5
	Total Lumbar, 33% group	BMD	1.108	0.03	1.129	0.03			0.7
	Total Lumbar, 66% group	BMD	1.055	0.03	1.073	0.04			0.51
	Total Lumbar, 138% group	BMD	1.068	0.03	1.087	0.04			0.54
Chen et al. (2017) [22]	Descending stair walking	BMD	–	–	–	–	15	*p* < 0.05	1.16

^a^Cohen’s *d* values represent magnitude of change of the variables (BMC, BMD) due to eccentric exercise intervention

*BMD* Bone mineral density (g/cm^2^), *BMC* Bone mineral content (g)

Only those intervention sites which showed significant effects of eccentric exercise are presented in this table

### Young adults

Five of the seven studies found in our search fit into the young adult population. ESE intervention in these studies ranged from 12 weeks to 20 weeks. In an early study examining the effects of 18 week ESE training on bone, authors [20] found that there were no significant differences between pre and post total hip BMD in a sample population of 16 females (20.8 ± 1.17 years of age). However, there were significant increases in the mid-femur BMD following ESE and not CSE training. These findings were partially supported by other studies examining the effects of ESE-only training on bone in young adults finding significant, site-specific BMD increases in the ulnar [32], total femur [19], and total lumbar spine [18]. The training period of ESE was 20 weeks for increased BMD at the ulnar [32] and total femur [19], while the beneficial effects on BMD at the total lumbar spine [18] was noted only in 12 weeks. Additionally, positive changes were reported in BMC of the ulna [32] and total forearm [19] due to ESE. In contrast, another study reported no changes in BMD following a 16-week ESE-only exercise protocol [21] but led to positive changes in BMC of the lumbar spine [21]. Finally, decreased BMC of the distal tibia in a 20 week ESE training study also has been reported in young adults [19].

### Middle-aged and older adults

In middle-aged and older adults, there is a lack of studies investigating the effects of ESE intervention on bone. Our search found only one middle-aged adult study published in 1998 [31] and one older adult study published in 2017 [22]. Alfredson et al. [31] had a sample size of 14 individuals with a mean age of 44.2 years (SD = 7.1) who underwent ESE intervention for 12 weeks. The ESE training included placing the injured ankle in plantar flexion and lowering the heel below the lever. To keep this task ESE only, participants used the non-injured foot to move the injured foot back to the starting position. The study required participants to exercise and increase their load at home. Interestingly, while the study conducted by Alfredson et al. [31] met the criteria of an eccentric training intervention; the pre-intervention and post-intervention values did not come from the same study population. Although the study groups that were assessed for BMD values were similar with respect to age, pathology, and symptoms, they were comprised of different populations. Thus, due to the use of a distinct set of individuals for pre- versus post-intervention BMD values, we could not report effect sizes for this study in Table 3. Notably, Alfredson et al. [31] reported the effects of their ESE intervention on BMD at the calcaneus with a coefficient of variation of ~5% using Dual-Energy X-ray Absorptiometry (DXA) in their laboratory.

Chen et al. [22] recruited a sample population of 30 older females with a mean age of 66.4 years of age (SD = 6.8) to participate in an intervention of either ascending (CSE) or descending (ESE) stair walking for a period of 12 weeks. For the ESE only, the descending stair walking group participants were transported from the bottom of the stairs to the top by elevator. The reverse procedure was used in the CSE-only group to maintain CSE only. The study found significant increases in BMD in the descending or ESE group only with a 6.1% increase in BMD from baseline to post-training at 12 weeks.

## Discussion

To our knowledge, this is the first review article to report the effects of ESE on BMD and BMC in humans. The main finding of our review paper is that evidence regarding beneficial effects of ESE on BMD and/or BMC in young, middle-aged, and older adults is inconsistent and variable. We also noticed a site-specific effect of ESE on BMD/BMC with skeletal sites of lower extremity [19, 20, 22] responding somewhat more favorably to ESE than skeletal sites of the upper extremity [32]. Interestingly, existing studies show small, yet statistically significant site-specific changes in BMD in relatively short intervention periods due to ESE. The duration of interventions, use of concentric 1-RM to establish ESE protocols, using different ESE protocols, small sample size, and non-homogeneity of the study participants in all the studies may explain the marked variability in the degree of beneficial effects of ESE on bone. Our review found the effect of ESE on BMD to be highest in older adults [22]. However, there was only one study using ESE in older adults [22]. Furthermore, only the studies involving middle-aged and older adults used closed kinetic chain exercise [22, 31] versus open kinetic chain exercise in all the other 6 studies included in our review article [18–21, 32]. It can be postulated that using novel eccentric maximum strength testing strategies for designing ESE protocols could produce greater mechanical loading on bone and thus translating to greatest benefits, specifically, in older adults. Notably, lack of any report of injuries during ESE protocol in these studies demonstrate the safety of exercising using ESE.

Indirect mechanical loading on bone from muscle contractions generates potent anabolic signals on bone [1] and thus drives skeletal adaptations [2, 3]. Direct mechanical loading on bone also induces anabolic signals for enhanced osteogenesis [9]. It is well-established that greater mechanical load leads to increased positive effects on bone density and strength [33]. Specifically, the overall magnitude of the load is a critical factor dictating skeletal adaptation [1, 9]. For example, when compared to recreational athletes, weightlifters have been shown to have significantly different BMD measures, such that the weightlifters’ BMD was greater than that of recreational athletes [34]. A combination of effects of direct and indirect mechanical loading on bone in weightlifters can explain their enhanced BMD. Typically, 70-85% of the maximum load that a muscle can lift concentrically, that is during the shortening phase, should be used in ‘conventional CSE’ to achieve beneficial effects on BMD to prevent osteoporosis [35]. Notably, this large magnitude of heavy stress may impose safety risk [16].

High stretch forces created during ESE exert significant mechanical loading on the skeletal system and thus could prove anabolic stimuli to bone. This was corroborated by one of the included studies which reported greater positive changes in BMD of the mid femur which performed ESE versus the contralateral leg which exercised with CSE in young adults [20]. A greater amount of connective tissue and increased passive stiffness helps to decline loss in eccentric strength with aging [23]. In fact, increased connective tissue and passive stiffness could increase the ability to produce passive mechanical strength and thus overall muscle eccentric strength with aging [36]. Furthermore, there is evidence that ESE results in a high force/strongly bound state of muscle cross-bridges during stretching resulting in the production of high forces during ESE [37]. Thus, ESE provides an interesting avenue to create a greater anabolic stimulus at lower stress for improving skeletal status in older adults. Importantly, ESE can easily be translated to include closed kinetic chain exercises and thus create direct and indirect mechanical loading on bone for an enhanced osteogenesis. In fact, recent advancements in technology have allowed rehabilitation scientists to design ESE-based programs in populations such as individuals with chronic conditions [38]. However, there is very little literature on the ESE effect on BMD or measures of bone strength in older adults.

According to the Mechanostat model, [1] mechanical load imposed on the skeletal system leads to adaptation in its mechanical properties, such as density and strength to meet new mechanical demands placed on it. Various mechanisms such as shear stress, direct strain, and pressure on osteocytes by increased fluid flow in the lacunar-canalicular network due to direct mechanical loading [9] are thought to generate the osteogenic signal for the skeletal system. These mechanisms are also referred to by the term ‘mechanotransduction’. Mechanotransduction also involves local growth factors and muscle turnover proteins such as increased insulin-like growth factor-1 which can stimulate osteogenesis [10]. Aging is associated with loss in the lacunar-canalicular network, especially in females [39]. This may explain, in part, the reduced anabolic potential of mechanical loading on the bone with aging.

**Fig. 3:**
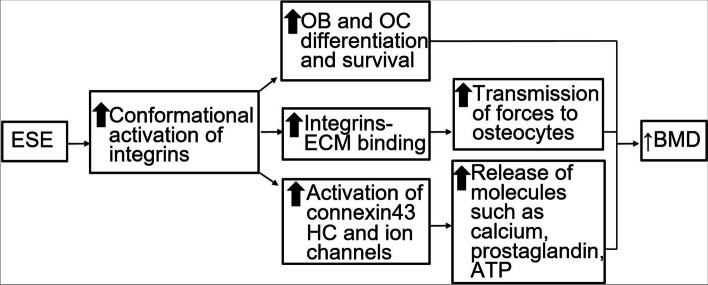
Conceptual diagram; ESE, Eccentric strengthening exercise; OB, Osteoblast; OC, Osteocyte; ECM, Extracellular matrix; HC, Hemichannels; ATP, Adenosine tri phosphate; BMD, Bone mineral density

A recent review article [40] showed that integrin-mediated cell-matrix interactions lead to a cascade of signaling pathways eventually resulting in increased osteoblast differentiation and survival. Integrins also play a critical role in osteocyte-induced mechanotransduction [41]. Specifically, osteocytes sense mechanical loading at integrin attachment-sites [42] and an increased expression of integrin signaling pathways reduces the rate of osteocyte apoptosis, thus augmenting osteogenesis [43]. Moreover, ESE produces high stretch forces which lead to increased expression of integrins [41]. Activation of integrins induces conformational changes and these effects on integrin lead to transmission of high stretch forces which can result in high mechanical loading on skeletal tissue [41]. Moreover, these integrin-mediated transmissions of high stretch forces can open mechanosensitive cation channels which promote osteogenesis by increasing intracellular calcium levels [44]. Additionally, the unique production of local and osteogenic factors which are anabolic to bone occurs due to high stretch forces, while integrins interact with extracellular matrix proteins to increase osteoblast differentiation [45] and osteocyte survival [41]. Thus, integrin-mediated mechanotransduction may be critical for maintaining or increasing BMD with aging as there is a greater expression of integrins with aging [46]. A conceptual framework showing potential mechanisms by which ESE can potentially produce anabolic and anti-catabolic effects on the skeleton is shown in Fig 3.

We were also interested to examine other factors that could explain potential of ESE to positively influence BMD as noted in our review. Prior evidence shows that increased BMD due to ESE could also be dictated, in part, by an increase in bone metabolism. For example, evidence shows that a single bout of eccentric contractions can increase bone formation markers such as osteocalcin and bone resorption markers such as cross-linked N-telopeptide of type I collagen [47]. It needs to be noted that the subacute effects of ESE can lead to differential bone formation and resorption response as noted by Huang et al. [48] Increased bone formation but lower bone resorption have been reported due to ESE in young populations [48]. Moreover, the positive effects of ESE on BMD may be site-specific. Support for site-specific benefits is found in previous reports of increased mid-femur BMD in response to ESE training of the knee extensors [20]. This is not surprising because force generation by eccentric contraction is greater by 20-30% versus concentric contraction, and thus can potentially mechanically load the skeletal system to a greater degree compared to traditional concentric forms of mechanical loading. Furthermore, these effects may be compounded in older adults because of lower loss of eccentric strength versus concentric strength with aging [23, 36].

Thinking from a ‘researcher’s mind and clinician’s heart’ approach, we wanted to examine the clinical feasibility of applying ESE in older adults. We think that the low metabolic cost of ERE contraction should propel it to the forefront of skeletal rehabilitation for older adults, especially for individuals with osteopenia/osteoporosis. The high stretch forces of eccentric contractions place lower metabolic demand than high muscle shortening forces [49]. Indeed, one of the main advantages of ESE is its markedly lower cardiometabolic cost [15]. Lower peak heart rate, systolic blood pressure, cardiac output, cardiac index, pulmonary ventilation, rate of perceived exertion, and respiratory exchange ratio have been reported with eccentric versus concentric contraction at comparable work levels in young and older populations [15, 27, 49, 50]. Lower cardiometabolic cost of eccentric versus concentric contraction may be explained by the lower muscle activation requirement needed to produce a comparable amount of force achieved by concentric contraction [51, 52]. Data exists showing that stretch forces during eccentric contraction create a lower requirement of Adenosine Tri Phosphate (ATP) to detach the myosin head from actin [37]. Moreover, the eccentric contraction has lower metabolic demand and has up to two times greater metabolic efficiency than concentric contractions [53]. A greater metabolic efficiency (reported as a lower requirement of ATP utilized per unit of work performed by muscle) has been reported with eccentric contractions versus concentric contractions [53]. A lower metabolic demand during eccentric contraction would not demand a significant increase in oxygen supply and thus could potentially lead to creating conditions for greater exercise or physical activity. Thus, ESE may hold special utility for populations with low exercise tolerance.

It is critical that we understand a clear difference between ESE and eccentric damage/injury [54]. Eccentric contractions have been a classical model of examining mechanisms of muscle damage [55–57]. This is because many studies have used maximal eccentric contractions to create skeletal muscle injury models and did not involve any element of progressive ESE [57]. Typically, the maximal eccentric contractions produced in those studies were supraphysiological and cannot be created in daily lives. Evidence has been accumulating that using the repeated bout effect while designing and implementing ESE is safe [58, 59] and associated with acceptability in patient populations [60]. Further, using the repeated bout effect during ESE training limits delayed onset of muscle soreness [60, 61].

### Strengths and weaknesses of the studies

Skeletal benefits of ESE were achieved in a short time frame which was a major strength of their intervention. For example, Miller et al. [32], Nickols-Richardson et al. [19], Hawkins et al., [20] and English et al. [18] reported bone adaptation results in relatively short time periods (≤ 20 weeks), however the effect sizes for related increases seen were small (Table 3). Miller et al. [32] and Nickols-Richardson et al. [19] both employed a 20-week ESE intervention during which both investigators reported increases in BMD or BMC. Hawkins et al. [20] reported changes in BMD in 18-weeks, Chen et al. [22] in 12 weeks, and English et al. [19] in only 8-weeks.

In addition, it is important to acknowledge the effect size relative to the p-values reported in each manuscript. Effect size in the current work was calculated as the “magnitude of the difference due to the intervention only” [29]. While the *p*-value is recorded and presented to the reader in order to show whether there is a statistical difference between groups, effect size has been calculated in order to show a more “substantive significance” [29]. Calculated effect sizes range from small to large but tend to remain in the moderate range for a majority of the studies (Table 3).

It can be postulated that the variance of results previously shown in ESE-only exercise studies can be partly explained by the specificity of the training protocols of each study (Table 2). No two studies employed the same training method, nor the same set to repetition ratio. Interestingly, even with individualized approaches to training, the one point of commonality was that each protocol was developed based on the subject’s CSE maximum, rather than an ESE maximum. While testing procedures for measuring a CSE 1-RM are well documented and widely available from established entities such as the American College of Sports Medicine and the National Strength and Condition Association, using a CSE 1-RM to determine training weight and progression may be considered a limitation in this pool of literature since ESE contraction could produce greater force than CSE contraction [33]. However, considering the publication dates of these studies, the ability of the investigators to test ESE 1-RM may have been difficult or an unreliable measure for which to base training protocols.

Although high stretch forces can induce unique cellular and molecular signaling resulting in increased BMC and BMD; studies used in our review article comprised of ESE utilizing open kinetic and closed kinetic chain. It is well known that mechanical loading interacts with muscle contraction forces during closed kinetic chain versus only muscle contraction forces act on bone during open kinetic chain. Interestingly, the greatest effect of ESE was observed in the study which comprised of closed kinetic chain exercise [22]. Thus, we do not know if the anabolic effect of ESE on bone is dictated differentially or as an interplay of muscle lengthening contraction versus mechanical loading on bone. Further, if these mechanisms are affected by sex or aging is unknown.

The results may also be partially explained by the method by which BMD or BMC was obtained. As with any form of measurement, varying types of BMD or BMC estimates have their own advantages and disadvantages. These studies have made use of three different techniques: DXA, mechanical response tissue analyzer, and quantitative ultrasound. Four studies used DXA [18–21], which is a well-established technique for high precision in measurement, reproducibility, with minimal amounts of radiation [34]. However, it is largely influenced by obesity and can be affected by both intrinsic and extrinsic artifacts [62]. Miller et al. [32] designated mechanical response tissue analyzer as its method of measurement as this technique is a noninvasive means of measuring long bones in vivo [32]. This technique uses the bone’s response to low-frequency vibration generated by a mechanical shaker through a probe placed on the participant’s skin [32]. Since vibration transmission is site-specific and can be attenuated differentially based on body biomechanics or composition [63], mechanical response tissue analyzer output cannot be compared with the DXA technique. The third method of measurement addressed in this review is quantitative ultrasound used by Chen et al. [22]. Ultrasound has the benefit of being portable and emitting no ionizing radiation. Conversely, ultrasound is highly operator dependent making reproducibility more difficult, [64, 65] specifically as related to quantifying therapeutic effectiveness [65].

### Strength and limitations of our review

One of the strengths of this review is the quality appraisal of studies. The PEDRO scale was used to conduct a quality assessment of studies reported in this review article. Effect sizes are also reported to show the effect of various ESE interventions on BMD and BMC. Although we have not reported weighted effect sizes based on site-specific effects, an overall effect size provides us a framework to design an evidence based ESE program for skeletal rehabilitation. The results from our study can also be used to design effective randomized controlled trials to assess the skeletal effects of eccentric training. Limitations of this paper include inclusion of only randomized controlled trials, as well as the use of only two output measures related to bone status: BMD and BMC. We used only BMD and BMC for a focused review. Moreover, besides BMD and BMC, there is a lack of consistency in reporting other outcome measures related to bone status such as bone formation and bone resorption markers, bone architecture, and bone strength. Notably, BMD and BMC are robust measures strongly related to bone strength [66]. Due to limited data published on the effects of ESE on BMC and BMD, it is unknown if the effect of ESE on BMD and BMC is sex dependent.

## Conclusion

Overall, our study shows that ESE has some potential to increase BMD and BMC in young, middle-aged, and older adults. However, there is large variability in ESE dosage and the administrative techniques of ESE among the published studies. Specifically, it is unknown if the effect of ESE on bone can be affected by the technique of utilizing different modes such as open kinetic chain vs closed kinetic chain exercise. Whether BMD and BMC effects of ESE are dictated by mode, dosage, age, or sex remains unknown and needs further investigation.

## Data Availability

Not applicable.
