# Correction: Skeletal effects of eccentric strengthening exercise: a scoping review

**DOI:** 10.1186/s12891-023-06826-8

**Published:** 2023-08-31

**Authors:** Harshvardhan Singh, Bethany A. Moore, Roshita Rathore, William R Reed, William R. Thompson, Gordon Fisher, Donald H. Lein, Gary R. Hunter

**Affiliations:** 1 Department of Physical Therapy, University of Alabama at Birmingham, Birmingham, AL US; 2 Department of Nutrition Sciences, University of Alabama at Birmingham, Birmingham, AL US; 3 Department of Physical Medicine and Rehabilitation, Heersink School of Medicine, University of Alabama at Birmingham, Birmingham, AL US; 4 Department of Physical Therapy, Indiana University, Indianapolis, IN US; 5 Department of Kinesiology, University of Alabama at Birmingham, Birmingham, AL US

***Correction: BMC Musculoskelet Disord 24, 611 (2023)***

10.1186/s12891-023-06739-6

Following the publication of the original article [1], the authors corrected the blinded data (XXXXXXXX) in the third sentence of subsection ‘Search strategy’ under ‘Methods’ section.

Incorrect version:

We also consulted a research reference librarian who works at XXXXXXXX to verify the article list using the same search terms.

Correct and revised version:

We also consulted a research reference librarian who works at the University of Alabama at Birmingham to verify the article list using the same search terms.

The original article [1] has been updated.
